# 259. Impact of a Multiplex Meningitis and Encephalitis PCR on the Management of CNS Infections in Adults and Children, Houston, Texas

**DOI:** 10.1093/ofid/ofab466.461

**Published:** 2021-12-04

**Authors:** Elizabeth A Aguilera, Shelby Simar, Susan H Wootton, Rodrigo Hasbun, Rodrigo Hasbun

**Affiliations:** 1 The University of Texas Health Science Center at Houston, Houston, TX; 2 University of Texas Health Science Center, Houston, TX; 3 McGovern Medical School at the University of Texas Health Science Center at Houston, Houston, TX

## Abstract

**Background:**

The majority of adults and children with meningitis and encephalitis have unknown etiologies fostering universal admission, prolonged length of stay, empirical antibiotic and antiviral therapies. A qualitative multiplexed nucleic acid-based in vitro diagnostic test (FilmArray) is helping clinicians identify organisms and improve clinical outcomes.

**Methods:**

Patients presenting between July 5, 2018, to April 26, 2021, with meningitis or encephalitis, cerebrospinal fluid (**CSF**) with WBC >5 cells/mm^3,^ and leftover CSF were available for testing. All CSF specimens underwent testing with the Biofire® Film Array Meningitis Encephalitis (**FAME**) panel. This multiplex PCR tool utilizes 0.2 ml CSF sample to identify the presence of 14 viral, bacterial and fungal pathogens in 1 hour.

**Results:**

Of 5291 CSF specimens screened, 285 (5.3%) met the criteria for meningitis or encephalitis and underwent FAME testing. The majority were adults (240, 84.2%), male (147, 51.5%), White (111, 38.9%), immunocompetent (213, 74.3%) and (212, 74.3%) had meningitis presentation. Median age was 41 years (0 mo-100 yrs) and (283, 99.2%) were admitted to the hospital. Median duration between CSF collection and viral PCR result as 13 hrs for Enterovirus, 31 hrs for HSV, and 57 hrs for VZV. The FAME panel detected a pathogen in 103 patients (36.1%) of whom 76 (73.7%) had a viral etiology. 166 (58.2%) patients were discharged with unknown etiology of whom FAME was positive in 24 (14.4%) [VZV (37.5%), HSV2 (16.6%), Enterovirus (16.6%), *Haemophilus influenzae* (8.3%), HHV6 (8.3%), *Streptococcus pneumoniae* (8.3%) and HSV1 (4.1%)]. The addition of the FAME panel to the standard of care evaluation increased the known etiologies from 119 (41.7%) to 197 pathogens (69.1%), (p< 0.05). 52 patients (18.2%) underwent a repeat LP (30, 57.6% done for additional testing). Empirical antibiotic and antiviral therapy were given to 89% and 63% of patients, respectively.

**Conclusion:**

A rapid multiplex PCR decreases the proportion of unknown etiologies and has the potential to decrease length of stay and the use of empirical antibiotic and antiviral therapies. Testing with the FA ME panel resulted in pathogen detections not previously recognized and for which treatment is recommended.

**Disclosures:**

**Rodrigo Hasbun, MD, MPH**, **Biofire** (Speaker’s Bureau) **Rodrigo Hasbun, MD, MPH**, Biofire (Individual(s) Involved: Self): Consultant, Research Grant or Support

